# Emergency department patient satisfaction survey in Imam Reza Hospital, Tabriz, Iran

**DOI:** 10.1186/1865-1380-1-2

**Published:** 2011-01-27

**Authors:** Hassan Soleimanpour, Changiz Gholipouri, Shaker Salarilak, Payam Raoufi, Reza Gholi Vahidi, Amirhossein Jafari Rouhi, Rouzbeh Rajaei Ghafouri, Maryam Soleimanpour

**Affiliations:** 1 Emergency Medicine Department, Tabriz University of Medical Sciences, Daneshgah Street, Tabriz-51664, Iran; 2 Department of Community and Health Medicine, Orumia University of Medical Sciences, Orumia-57147, Iran; 3 Department of Public Health, National Public Health Management Center (NPMC), Faculty of Health and Nutrition, Tabriz University of Medical Sciences, Tabriz-51664, Iran; 4 Member of Student Research Committee, Tabriz University of Medical Sciences, Tabriz-51664, Iran

## Abstract

**Introduction:**

Patient satisfaction is an important indicator of the quality of care and service delivery in the emergency department (ED). The objective of this study was to evaluate patient satisfaction with the Emergency Department of Imam Reza Hospital in Tabriz, Iran.

**Methods:**

This study was carried out for 1 week during all shifts. Trained researchers used the standard Press Ganey questionnaire. Patients were asked to complete the questionnaire prior to discharge. The study questionnaire included 30 questions based on a Likert scale. Descriptive and analytical statistics were used throughout data analysis in a number of ways using SPSS version 13.

**Results:**

Five hundred patients who attended our ED were included in this study. The highest satisfaction rates were observed in the terms of physicians' communication with patients (82.5%), security guards' courtesy (78.3%) and nurses' communication with patients (78%). The average waiting time for the first visit to a physician was 24 min 15 s. The overall satisfaction rate was dependent on the mean waiting time. The mean waiting time for a low rate of satisfaction was 47 min 11 s with a confidence interval of (19.31, 74.51), and for very good level of satisfaction it was 14 min 57 s with a (10.58, 18.57) confidence interval. Approximately 63% of the patients rated their general satisfaction with the emergency setting as good or very good. On the whole, the patient satisfaction rate at the lowest level was 7.7 with a confidence interval of (5.1, 10.4), and at the low level it was 5.8% with a confidence interval of (3.7, 7.9). The rate of satisfaction for the mediocre level was 23.3 with a confidence interval of (19.1, 27.5); for the high level of satisfaction it was 28.3 with a confidence interval of (22.9, 32.8), and for the very high level of satisfaction, this rate was 32.9% with a confidence interval of (28.4, 37.4).

**Conclusion:**

The study findings indicated the need for evidence-based interventions in emergency care services in areas such as medical care, nursing care, courtesy of staff, physical comfort and waiting time. Efforts should focus on shortening waiting intervals and improving patients' perceptions about waiting in the ED, and also improving the overall cleanliness of the emergency room.

## Introduction

Satisfaction is an important issue in health care nowadays. The emergency department (ED) is considered to act as a gatekeeper of treatment for patients. Thereby, EDs must achieve customer satisfaction by providing quality services.

According to Trout, statistics show that the number of ED clients is steadily increasing. This is an indicator of the importance of planning quality services based on the needs of these patients. In order to plan successfully, understanding the views, needs and demands of clients is an essential step. A common tool to improve the quality of care in the ED is to conduct a client satisfaction survey to clearly explore the variables affecting the satisfaction level and causes of dissatisfaction. Clients' satisfaction is a key component in choosing an ED for receiving services or even for recommending it to others [1].

Although it may seem impossible to keep all clients satisfied, we can achieve a high level of satisfaction by working on related indicators and trying to improve them [2].

Studies from other countries indicate that using the results obtained from satisfaction surveys can have a profound effect on the quality of services [3-5].

In this study, we examined the satisfaction level of clients presenting to the ED of Imam Reza Teaching Hospital, which is one of the leading EDs in northwest Iran, with approximately 65,000 admissions per year.

## Methods

This cross-sectional study with descriptive and analytical aims was conducted in 2008, and the participants included our ED clients. Taking into account that busy work hours, shifts, personnel, different providers, day of the week and type of client complaint have an effect on satisfaction level, we selected our sample randomly considering the above factors.

The sample distribution of the population consisting of 500 ED clients was carried out using accidental quota sampling. In the study period, the number of clients was 1,630 in 1 week. In the morning shift 578, in the evening shift 611 and in the night shift 410 clients were seen at the ED. Considering the fact that 500 people were selected as the sample population, the quota for the morning, evening and the night shift was 35.5%, 37.5% and 25.2%, respectively. In the study period, for selecting the people in each shift, random numbers were used to choose the individuals for the study. The questionnaires were given to the patients after they agreed to complete them. No evidence of unwillingness was detected, and all consented to cooperate.

The satisfaction questionnaire of the Press Ganey Institute, which is being used in most American hospitals with more than 100 beds, was implemented in this survey. The literature indicates that 49 EDs in general hospitals and a 2002 study in Milwaukee, Wisconsin, also used this questionnaire [3,4]. This institute has reported the status of patient satisfaction with visits to the ED every year since 2004 using collected data from all 50 states in the US [6].

In our study, we used this questionnaire with minor modification of some items because Iran's admission, visit and discharge processes are somewhat different from those in the US. The items we added to the questionnaire are the following:

1. The literacy status and educational background of the interviewee

2. Satisfaction of the interviewees with the ED security guards' courtesy and behavior

The two items "Personal Issues" and "Access to Care" were completely omitted from the original questionnaire.

We validated the revised Press Ganey questionnaire by distributing it to ED specialists and academic members to confirm its content validity.

The study used the highly valid and reliable Press Ganey questionnaire consisting of 30 standard questions organized into four sections:

1- Identification and waiting time

2- Registration process, physical comfort and nursing care

3- Physician care

4- Overall satisfaction with the emergency department.

Interviews were conducted by research team members. The language used in preparing the questionnaire was Farsi, which is the official language of the country. The interviewers did not wear uniforms or badges. After introducing the objectives of the research to the patients and learning about their willingness to participate, the interviews were started. Subjects were interviewed once they exited the ED, both those who were going to be hospitalized in a ward or who were being discharged from the ED.

In this study, the waiting time before the first examination of the patient was also measured. The exact time of the patient's arrival was recorded in his/her medical records upon their arrival, as was the first examination by the physician. According to these recorded times, the minutes the patient had spent waiting could be determined.

In order to reduce an interview bias, the interviewers were oriented in a session by academic members of the ED with respect to unifying their communication and the process of interviewing the patients. The collected data were analyzed using SPSS version 13. Nominal and ordinal scale data were reported as absolute and relative frequency, and normally distributed data were presented as means ± standard deviation. To determine any differences between groups, data were analyzed by X^2 ^test; the odds ratio and 95% confidence interval were calculated to determine the relationships between the variables examined. P < 0.05 was considered to be statistically significant.

## Results

Analysis of the data indicates that 500 clients out of the total number of clients referred to the ED agreed to participate in the study. Demographic characteristics of the participants are fully indicated in Table 1. Because some questionnaires were not fully answered by the participants, a small proportion of the data was regarded as missing.

**Table 1:** Demographic characteristics.

Population-specific demographic	Percent
**Gender**	
Female	40.8
Male	59.2
**Level of education**	
License & high education	14.3
Technician	9.5
Diploma	25.7
Under diploma	36.2
Illiterate	12.5
**Time of visit**	
Morning	35.5
Evening	37.5
Night	25.2
Missing	1.8
**Patient's first visit here**	
Yes	37.3
No	62.7
**Who has completed the questionnaire**	
Patient	9.4
Another one	89
Missing	1.6
**Living location**	
Urban	82.5
Rural	17.3
Missing	0.2
**Patient's disposition**	
Discharge	60.6
Admission	18.9
Expired	0.7

The data also indicate that 9.5% of the participants were patients, 89% were their relatives and 1.6% of them did not answer the questions completely. Also, 37.5%, 35.5% and 25.2% of the interviewees were admitted to the ED in the evening, morning and night shifts, respectively. Only 37.3% of them were using our ED services for the first time.

The majority of the subjects we studied were male (59.2%), and 40.8% were female. One third were living in Tabriz, which is a major city and provincial center in Iran. The minimum age of subjects was 12 years and the maximum 92 years, with an average value of 43.9 years.

Further analysis of the data revealed that in terms of the literacy and academic background of the interviewees, the largest group (36.2%) comprised those who were either illiterate or had left school before getting their high school diploma. The least frequently represented group (9.5%) was that with participants holding an associate degree (a degree equal to college completion). In other words, 50% of the subjects had received an education below the level of a high school diploma. The data also show that 60.6%, 18.4%, 18% and 0.7% of the patients who were admitted to the ED were discharged, hospitalized, referred or died, respectively. We need to mention the 1.8% of the population here that was regarded as missing. This study reveals that the waiting time (WT) for the first visit to emergency medicine residents or specialists was 24.15 min, with a maximum of 35 min and minimum of 1 min.

For the association analysis between waiting time and satisfaction levels, *P *= 0.03 indicates that those with longer WTs were dissatisfied. Table 2 shows the satisfaction level of clients in regard to 20 items of the questionnaire.

**Table 2:** Satisfaction level of clients in regard to 20 items of the questionnaire.

Question	Very poor	Poor	Fair	Good	Very good
Courtesy of staff in the registration area	4.5	2.7	16.3	2.7	4.5

Comfort and pleasantness of the waiting area	8.7	10	25.3	21.5	34.5

Comfort and pleasantness during examination	12.5	3.4	14.6	14.3	55.2

Friendliness/courtesy of the nurse	6.1	2.9	13	17.9	61

Concern the nurse showed for doing medical orders	6.2	3.8	12.9	28	56.3

Courtesy of security staff	6.8	2.3	12.7	18.6	59.6

Courtesy of staff who transfer the patients	11	4.3	11.5	19.6	53.6

Length of wait before going to an exam room	16.8	9.4	15.6	17.3	40.9

Friendliness/courtesy of the care provider	4.9	2.2	10.4	16.7	65.8

Explanations the care provider gave you about your condition	8.6	7.8	16.4	16.4	50.8

Concern the care provider showed for your questions or worries	7	7	18.5	18.8	48.7

Care provider's efforts to include you in decisions about your treatment	17.8	8.7	13.2	14.3	46

Information the care provider gave you about medications	10	8.3	14.5	17.8	49.4

Instructions the care provider gave you about follow-up care	7.8	8.1	11.3	15.6	57.2

Degree to which care provider talked with you using words you could understand	6.9	5.1	15.2	13.3	59.5

Amount of time the care provider spent with you	9.3	10.9	15.4	15.7	48.7

Frequency of being visit by physicians	9.8	5.5	19.3	16.7	48.7

Overall cheerfulness of our practice	7.7	5.8	23.3	28.3	34.9

Overall cleanliness of our practice	14.5	7.7	19.8	29.3	28.7

Likelihood of your recommending our practice to others	10.9	7.5	16.6	27	38

Items with a high level of satisfaction included: physicians' courtesy and behavior with the patients (82.5%), security guards' courtesy (78.3%) and nurses' courtesy with the patients (78%).

The lowest level of satisfaction refers to the following items: care provider's efforts to get the patients involved in making decisions about their own treatment (26.5%), waiting time (WT) for the first visit (26.2%), and cleanness and neatness (22.2%).

The mean waiting time for the patients to be visited by a specialist was 24.15 min, ranging between 35 min as the maximum and 1 min as the minimum waiting times. The highest level of satisfaction with the ED was related to physicians' courtesy (83.1%), and the lowest level was related to service men's friendliness (15.4%). The participants also rated their overall satisfaction of care received during their visit as very high (35/9%), high (28.3%), average (23.3%), low (5.8%) and very low (7.8%).

Thus, the data indicate that overall satisfaction was 63.2%, although (13.6%) were dissatisfied. Once the patients themselves were interviewed, their satisfaction level was 60.6%. On the other hand, their relatives' satisfaction level was 63.2%. Also, 18.5% of patients and 13% of their relatives reported dissatisfaction. The difference in satisfaction rate between the two groups was statistically significant (*P *= 0.03).

In regard to work shifts, subjects' satisfaction with the morning, evening and night shifts were 62.4%, 64.3% and 63.3%, respectively. Their dissatisfaction levels were 12%, 12.7% and 14.3%, respectively. Although the overall dissatisfaction rate for the night shift was less than that for the other shifts, there was no meaningful statistical difference among the different shifts.

The data also indicate that living area, either urban or rural, showed no meaningful relation to satisfaction level.

The satisfaction levels in regard to the subjects' educational background were 45.7%, 51.5%, 53.7%, 76.3% and 65.8% for those holding bachelor degrees and above, associate degrees, high school diplomas, those under the high school level and those who were illiterate, respectively. Dissatisfaction levels among them were 23.9%, 9.1%, 13.7%, 9.1% and 18.4%, respectively. Data analysis shows that those with higher educational levels were more dissatisfied (*P *= 0.05). Once the subjects were asked whether they would recommend this ED to others or would refer to it again, 65% and 18.4% indicated that they would and would not, respectively.

## Discussion

Patient satisfaction is considered one of the important quality indicator(s) at the ED [1]. Measurement of patient satisfaction stands poised to play an increasingly important role in the growing push toward accountability among health care providers [3].

According to the report of Press Graney Associates (2009), the emergency department (ED) has become the hospital's front door, now accounting for more than half of all admissions in the United States [6]. This has placed considerable strain on many facilities, with the increasing demand for service - much of it inappropriate to the site of care - leading to long waiting times, crowded conditions, boarding patients in hallways, increased ambulance diversions, and highly variable care and outcomes [6].

Due to the fact that the ED is a unique department among other medical care services, understanding of the factors affecting patient satisfaction is essential [5].

Our survey, like similar studies, indicates that the general satisfaction of clients is high, although there are many unmet needs [7].

Findings indicate that 34.9% of the clients show very high general satisfaction with regard to ED performance. Further analysis of the data shows that 13.5% have low satisfaction. In total, 86.5% of the clients rated their satisfaction as above average.

The Press Ganey Emergency Department Pulse Report 2009 found that patient satisfaction rose in 2008, continuing a 5-year trend of improvement. This report, which represents the experiences of 1,399,047 patients treated at 1,725 hospitals nationwide between 1 January and 31 December 2008 in the US, reveals that overall patient satisfaction with the ED was 83.18% [6].

Our findings also indicated that there is an association between satisfaction and being the patient's relative, educational level, time of admission and resident area (rural or urban). However, further analysis reveals that except for the interviewees themselves (patients or their relatives) and their educational backgrounds as two factors, there is no meaningful association between other factors and satisfaction.

Patients' relatives were more satisfied with the ED than the patients themselves were, and the patient satisfaction level was lower in those with higher educational levels. Time of admission, gender difference and place of residence had no meaningful relation with satisfaction level. Patients who arrived in the emergency department between 2:00 p.m. and 8:00 p.m. reported higher satisfaction than those who arrived in the morning or overnight hours; however, there was no meaningful statistical difference among different times of the day. In the Press Ganey report the highest satisfaction with the emergency department was recorded in the morning hours. The influences of gender, race, educational level and place of residence on patient satisfaction were not assessed in this report [6]. Staffing patterns, patient volume and severity of the patient conditions may play a large part in these differences in satisfaction. In the night hours, waiting times may be on the rise as patient volumes have increased during the day.

The study by Hall and Press (1996) in the US shows that variables such as age and gender do not have a profound impact on satisfaction level. It also shows that an association exists between patients' satisfaction and the respect they receive from physicians and nurses during waiting times [5].

Aragon's study reveals similar results; overall satisfaction was equal despite gender [8].

Consistent with other research, our results demonstrated that patient gender does not materially influence ED patient satisfaction.

The findings of the study by Omidvari and colleagues at five large hospitals of the Tehran University of Medical Sciences were to some extent similar to our findings: 85.6% and 41.8% of clients showed satisfaction above average and very good, respectively. Those with higher education were less satisfied, but there was no significant relationship between marital status, occupation, gender, work shift and satisfaction level. It is also true that those who waited longer were less satisfied [9].

In another study in provincial teaching hospitals in Ghazvin, Iran, 94.4% of the clients were satisfied with hospital services. In total, 59% were satisfied with services provided in the ED. This study shows that a meaningful relationship exists between age, gender, education level and satisfaction [10].

A systematic review that was undertaken to identify published evidence relating to patient satisfaction in emergency medicine carried out by Taylor and Benger (2004) showed that patient age and race influenced satisfaction in some, but not all, studies [11].

The findings of our study revealed that the average time a patient waited to be seen by a specialist or a resident in emergency medicine was 24.15 min. There was an association with satisfaction level; those who waited longer were less satisfied (*P *= 0.03).

Hedge's study, which was conducted with 126 patients with an average waiting time of 13 min, showed similar findings; those who waited longer were less satisfied [12].

In another study in 2004 at Cooper Hospital in New Jersey, the satisfaction level was higher in those with serious illnesses or emergency needs. In this study they suggested that the reduction in average waiting time was an important factor to increase the satisfaction level [13].

Compared with similar studies, the waiting time in our study was not much more; however, it was the second dissatisfaction factor that was rated. On the other hand, items with a high level of satisfaction included: physicians' courtesy with patients, security guards' courtesy and respect, and nurses' respectful behavior with patients. The two important factors that influenced patient satisfaction seem to be the waiting time and staff service and courtesy.

Aragon's investigation indicates that overall service satisfaction is a function of patient satisfaction with the physician, with the waiting time and with nursing service, hierarchically relating to the patients' perception that the physician provides the greatest clinical value, followed by time spent waiting for the physician and then satisfaction with the nursing care [12]. In this regard, the literature provides ample evidence that satisfaction with waiting time, and nursing and physician care influences overall satisfaction with emergency room service and that these are key factors in the measurement of overall satisfaction.

A cross-sectional study in Turkey among 1,113 patients indicated that there was a profound association between the physicians' skills, friendliness or courtesy of physicians, the process of triage, information the care provider gave the patient about his/her illness and medications, the discharge process and satisfaction level. Lengthy waiting times had a direct relationship with patient dissatisfaction. On the other hand, reduction of waiting time had no effect on satisfaction level [14]. In the Press Ganey report (2009), patients who spent more than 2 h in the emergency department reported less overall satisfaction with their visits than those who were there for less than 2 h. Since much of the time in the ED is spent waiting - in the waiting room, in the exam area, for tests, for discharge - reducing waiting times should have a direct positive impact on patient satisfaction [6].

In another study in Turkey with 245 patients, lengthy waiting time and quality of ED services were the most important reasons for dissatisfaction and satisfaction of patients, respectively. The resulting belief was that patient satisfaction is an important indicator of quality of medical care service in EDs [15].

Findings of a study in teaching hospital EDs in Arak, Iran, indicate that admission wards and physician services receive 18 points out of 25 (72%) and 33 out of 45 (73%) in regard to patient satisfaction level. This study also demonstrated that there was a high dissatisfaction rate with the cleanness and suitability of public services and toilets [16].

In another study conducted in Iran, the satisfaction rate was as follows: medical and nursing care (78.6%), satisfaction with the environment (78.3%) and health status (68.8%). The majority of the sample (76.5%) was satisfied with the hospital EDs. Although the satisfaction level with quality services was considerably high, there was a substantial dissatisfaction with the availability of adequate facilities, physical environment, inpatient care and security staff courtesy [17].

Our study's findings reveal that a high satisfaction rate can be achieved by courtesy and respect shown to the clients by the staff. Communication skills seem to be an important factor in ED management and may improve patient satisfaction. A study in Hong Kong supports our belief that workshops on communication skills can improve doctors' abilities in this area with a corresponding increase in patient satisfaction and decrease in patient complaints concerning ED doctors [18].

Our findings also indicate that only 15.3 percent of the sample was dissatisfied with students' interventions in their treatment and examination process. Similar to other studies, our findings also showed that waiting time and the physical environment of the ED are among the factors causing much dissatisfaction and that they can be reduced by setting up a good triage system and trying to create a neat environment. The literature indicates that the comfort of the waiting room and cleanliness of the ED environment are also important patient satisfaction factors in the US: Those who rated the waiting room as "very poor" in comfort had dramatically lower overall satisfaction with their visit than those who rated the comfort of the waiting room as "very good" [6].

Moreover, a research study in Hazrat Rasoul Hospital (Tehran, Iran) revealed that by setting up a waiting room, using guide signs, admitting patients with a bedside form and having a member of the staff welcome clients raised the level of satisfaction considerably, from 49% to 83% in 2 years of follow-up [19].

In a similar study in 2004-2006, after an intervention moving the ED to a new location, establishing a quality management system, hiring ED specialists and experienced nurses and mechanizing the infrastructures, the satisfaction trend improved progressively in four stages from 59.7% to 64.2%, 71.4% and then 74.4% [20].

Thus, according to the findings of this study and similar ones, in order to raise the satisfaction level, EDs need to define their processes very clearly, especially those processes related to diagnosis and treatment, admission and discharge, and sorting emergency patients from acute cases admitted to the ED. On the other hand, EDs that cannot reduce waiting times can recover some patient satisfaction by improving the comfort of their waiting rooms. Hospitals can analyze their patients' comments to find ways to improve the comfort level. Simple things such as repairing the air conditioning or replacing the chairs may have a noticeable effect on the patients' perceptions of the ED.

In Tailor's study in Australia, it was evident that staff orientation with an educational film and workshop on how to communicate effectively with patients and having a nurse to explain the diagnostic and treatment processes to patients improved the patient's satisfaction levels [21].

Although the skill of health care providers and their friendliness and courtesy are important factors in patient satisfaction, effort should focus on shortening the waiting times as well as improving patients' perceptions about waiting in the ED. While longer waiting times increased patient frustration, it was not known whether differences in waiting time reflected actual differences in clinical quality. Patient perceptions of emergency department care quality were also much lower than perceptions of care quality at other ambulatory care providers, even for patients with similar waiting times.

## Limitations

There are some serious confounding factors in our study. We believe that evidence-based interventions can be adopted based on such survey data. However, the survey results might not be generalizable because of regional differences. We did not measure the time spent in the ED from patients' arrival until disposition. This seems to be another important factor that may have a noticeable effect on patient satisfaction. Patients with different presentations might have different satisfaction rates, and the severity of cases may influence satisfaction rates, e.g., people who are in a great deal of pain are likely to be dissatisfied.

There are different types of questionnaires to measure patient satisfaction. Which patient satisfaction measurement can be further integrated into an overall measure of clinical quality is unknown. Variation in measurement tools, however, hinders making patient satisfaction a reliable part of the quality equation. Data on patient satisfaction are currently collected by various entities for different purposes and at different levels in the health care system. The questionnaire used in our study is from the Press Ganey Institute. Another questionnaire that is commonly used in Europe is the questionnaire developed by the Picker Institute. The Picker questionnaire focuses on the patient care processes and can be used in similar studies.

## Conclusion

Our findings showed that in order to provide optimal ED services and win patients' satisfaction, research-based interventions are needed in areas such as clinical care processes, nursing services, staff behavior and treatment of patients, physical environment and waiting time. To make these improvements, institutionalizing quality management in health services is a must, and using its feedback in a systematic way can enhance efficiency and patient satisfaction with the ED.

## Competing interests

The authors declare that they have no competing interests.

## Authors' contributions

HS, RGV and RRG conceived of the study, participated in its design and coordination, and wrote the first draft of the manuscript. CG, AJR and MS developed the study design and contributed to manuscript preparation. SS and PR participated in the design of the study and performed the statistical analysis. All authors read and approved the final manuscript.
